# Editorial

**Published:** 1862-09

**Authors:** 


### Editorial.

### MEDICAL DEPARTMENT OF LIND UNIVERSITY.

The regular annual course of lectures in this Institution commenced on Monday evening, Oct. 13, 1862, with a general introductory lecture, by Prof. R. N. Isham. The lecture room was well filled with an appreciative audience, who listened to the lecture with much interest and pleasure. The lecture was well written, its sentiments strictly appropriate to the occasion, and well calculated to impress the mind of the student with a proper appreciation of the importance of his undertaking, and to inspire him with a laudable ambition to make himself an honor to the profession and a blessing to the community. At the close of the lecture, Prof. Johnson, president of the Faculty, explained such changes as had been made necessary on account of the continued absence of one or two members as surgeons in the army. The full course on general pathology and public hygiene will be given by Dr. Wing, one the members of the State Board of Medical Examiners, a true gentleman, a ripe scholar, and fully competent to do justice to the task he has undertaken. David Rutter, M.D., emeritus professor of obstetrics, will add to the interest in the department of midwifery and diseases of females, by giving one lecture per week throughout the term. In addition to the four regular clinics in the Mercy Hospital and two in the College Dispensary, Prof. Isham will give a full clinical course on syphilis and syphilitic diseases in the Marine Hospital, occupying every Saturday morning. The following is the complete programme for the present College term :—

JUNIOR COURSE.

| | MONDAY. | TUESDAY. | WED'SDAY. | THURSDAY. | FRIDAY. | SATURDAY. |
|---|---|---|---|---|---|---|
| 9 A.M. | Johnson, | Johnson, | Johnson, | Johnson, | Johnson, | |
| 10 A.M. | Wing, | Wing, | Wing, | Mahla, | Mahla, | |
| 11 A.M. | Hollister, | Hollister, | Hollister, | Hollister, | Hollister, | Mahla. |
| 12 M. | Jewell, | Jewell, | Jewell, | Jewell, | Jewell, | |

SENIOR COURSE.

| | MONDAY. | TUESDAY. | WED'SDAY. | THURSDAY. | FRIDAY. | SATURDAY. |
|---|---|---|---|---|---|---|
| 2 P.M. | Byford, | Byford, | Clinic, | Byford, | Byford, | Clinic. |
| 3 P.M. | Isham, | Isham, | Isham, | Isham, | Isham, | |
| 4 P.M. | Mahla, | Mahla, | Mahla, | Rutter, | Spafford, | |
| 5 P.M. | Davis, | Davis, | Davis, | Davis, | Davis, | |

Mercy Hospital Clinic, 8 A. M. Monday, Tuesday, Thursday, and Friday.
Marine " " 8 A. M. Saturday.
College, " " 2 P. M. Wednesday and Saturday.

---

"Is Alcohol Food?"—We find in a recent number of the *London Lancet*, a summary or abstract of a paper read at the Annual Meeting of the British Medical Association, Aug. 8th, 1862; by Thomas Inman, M.D., Physician to the Liverpool Royal Infirmary, on the above subject. The following are the conclusions of the author:—

1. Nature has provided in the salivary glands, the liver, and the lungs of every mammal an apparatus for converting all food, especially farinaceous, into alcohol; and we have no evidence that such conversion does not take place.

2. One form of alcohol or another is available for the support of life, and for restoration to health when no ordinary food can be or is digested.

3. Alcohol, after being taken, is incorporated with the blood, passes into the various tissues, and ultimately disappears, a small portion only passing away in the breath. We can say no more of bread, potatoes, or oatmeal porridge, a small portion of each of which passes out of the body with the fæces.

4. Alcohol, in the form of ale, porter, wine, etc., relieves hunger and quenches thirst simultaneously, and with a completeness that is not equalled by water, infusion of gentian, cayenne pepper, or by turpentine—*i. e.*, it does not act as water simply, or as a stimulant alone.

5. Wine, beer, etc., satisfy the appetite when taken alone, and act for the time as any solid food would do.

6. When alcohol is mingled with other food, a less amount of the latter suffices for the wants of the system than if water had been used as the drink.

7. The various forms in which alcohol is taken have as marked and specific effects as have animal and vegetable articles of diet.

8. Individuals have subsisted wholly upon one or the other of the various forms of alcohol in common use for periods of great length; and as it is illogical to conclude that they must have lived on air, without food, or on flies, like chameleons, the conclusion is irresistible.

What that conclusion is, it might be left for every thinking man to decide.

In regard to the first of these propositions, we find nothing in the paper or elsewhere to show why the author might not have said with equal propriety that, "Nature has provided in the salivary glands, the liver, and the lungs of every mammal an apparatus for converting all food, especially farinaceous, into" *soap* or *vinegar* as into "alcohol." It seems to be a simple assertion without the slightest positive proof for its support.

Neither is the negative assertion made in the closing sentence of the proposition true; for we have evidence in abundance, that no such conversion of food into alcohol ever takes place in the healthy state of the animal economy.

1st. The numerous rigid and elaborate analysis of healthy blood, made by different chemists, have entirely failed to detect any such substance as alcohol in that fluid, whether arterial or venous.

2d. There is nothing in the known composition or properties of the fluids furnished by the salivary glands, the liver, or the lungs calculated to produce vinous fermentation.

3d. The eating of any quantity of the *ferinaceous* food has never been known to produce any of the peculiar exhilerating effects of alcohol. Did Dr. INMAN ever see a man intoxicated from drinking simple syrup, or eating any quantity of sugar, starch, or gum?—And yet if "nature" has provided the digestive and pulmonary organs "for converting all food, *especially farinaceous, into alcohol*," why does not the son of Erin get just as *tipsy* at a suitable time after a breakfast of potatoes, as after a glass of whiskey?—The second and third propositions contain nothing that might not be said with equal propriety of any other valuable medicinal agent.

The intimation that only a small part of the alcohol taken, is ever eliminated from the system unchanged, though disproved by the recent experiments of LALLEMAND and others, yet if it was true, affords no proof that the retained alcohol acts as food for nourishing the tissues.

Opium, mercury, arsenic, etc., are also taken into the blood, and pass into the various tissues, and no one has yet demonstrated that all of these substances reappear unchanged in any of the secretions. But shall we therefore conclude that opium, mercury, and arsenic are equivalent to food?

Such a conclusion would be just as logical and philosophical as that involved in the third proposition of the author.

The assertion in the fourth proposition that "ale, porter, wine, etc., relieve hunger and quench thirst simultaneously, and with a completeness that is not equalled by water, is partly true and partly false. It is true that the addition of alcohol to the water will relieve the sense of hunger more than clear water; and so will opium, ether, chloroform, or any other agent capable of producing an anodyne or anesthetic effect on the nervous system. But that it will quench thirst more perfectly or for a longer period than pure water is contradicted by every day's observation; for certainly there is no class of persons who call for drink more frequently or more urgently than those who drink fermented or distilled liquors.

The fifth, sixth, and seventh propositions are of the same vague and unsatisfactory character as the preceding.

The assertion in the eighth proposition, that "Individuals have subsisted wholly upon one or other of the various forms of alcohol in common use, for periods of great length;" leaving it as a necessary inference that it must consequently have acted as food, is altogether unsatisfactory. How long does the author mean by "periods of great length?" Is it three weeks, three months, or three years? Again, "one or other of the various forms of alcohol in common use," is rather indefinite.

It is well known that most of the fermented drinks as they are commonly used, contain a small quantity of matter capable of acting as nourishment aside from the alcohol. Hence, if an individual supplied with those drinks should live longer than one supplied with pure water only, it would not prove that the alcohol either acted as food or was the cause of the prolongation of life. When Dr. INMAN will show by any well-conducted series of experiments, that a single drop of alcohol is actually produced or generated in any organ, structure, or secretion of the healthy human body; or that any quantity of alcohol taken into the system from without actually furnishes organic atoms for the nourishment of any structure, then he will have done something towards settling the vexed question whether "Alcohol is food." Alcohol is an agent possessed of well-known and easily recognized properties. Its presence in any of the fluids or solids of the human body is easy of detection by the proper tests. These fluids and solids have been examined and analyzed again and again, by the most eminent chemists and chemico-physiologists of all countries; but in no instance have they found a drop of alcohol, unless it had been previously introduced into the system from without. If "nature" had really "provided in the salivary glands, the liver, and the lungs of every mammal an apparatus for converting all food, especially farinaceous, into alcohol," would not some of these analytical investigators have detected, at least, traces of the existence of this well-known agent? And do not the uniformly negative results of all analysis, so far as regards the presence of alcohol afford *some* "evidence that such conversion (as food into alcohol) does *not* take place," in the living system? In fact, does not every intelligent student of physical science know, that alcohol is never the product of any living vital action in any part of either the animal or vegetable kingdoms of the universe; but is solely the product of destructive decomposition or fermentation taking place in inanimate or dead matter?

In the name of science and common sense, we ask when there will be an end of such loose, illogical, and dogmatic conclusions as those put forth by Dr. INMAN, concerning the conversion of food into alcohol or alcohol into nutritive matter for the tissues? In the popular mind, alcohol has long been the guardian angel with the flaming sword turning every way to guard the tree of life. According to the popular notions, it is equally efficacious in protecting the human system against the cold of winter and the heat of summer; against the dry and scorching air of the desert and the fogs and vapors of the marsh, etc., etc.; and, at the present time, it has well nigh reached the same ubiquitious position in the professional mind. We are told to give it in the water we drink to prevent disease; to give it in the active phlegmasia as acute pneumonia; to give it still more largely in the low typhoidal forms of disease; to give it as a medicine; to give it as food; to give it as a preventive before disease begins; to give it as a curative when disease exists; to give it as nourishment after the disease has left; and, finally, Dr. INMAN gravely tells us that the digestive organs simply constitute an apparatus for converting *all food* into alcohol. What a happy thing it would be for the human race if, taking this proposition of Dr. INMAN to be true, that all food, and especially farinaceous, is converted into alcohol by the digestive organs, they would act upon the legitimate practical inference, that it would do just as well to eat a dish of rice or potatoes or drink a cup of molasses, as to drink a glass of wine or whiskey.

---

RESIGNATION OF ARMY SURGEONS.—We learn that several of the more eminent and experienced surgeons in the volunteer part of the army have resigned. Among them we notice the familiar names of Dr. CROSBY, of New Hampshire, and Dr. D. PRINCE, of this State. Both had occupied the position of Brigade-Surgeon; and both had maintained a high reputation on the field of active service. They will be cordially welcomed by their hosts of friends at home; yet our army can hardly afford to lose the services of such men at the present time.

---

ARTIFICIAL LIMBS FOR SOLDIERS.—The last Congress appropriated a fund for supplying artificial limbs to maimed soldiers; and the surgeon-general appointed a commission to devise a proper method of expending the same. This commission, consisting of Drs. VAN BUREN, GROSS, J. M. WARREN, and SATTERLEE, has decided to allow each patient fifty dollars for a lower and twenty-five dollars for an upper extremity. The same commission selected as manufacturers of artificial limbs, Drs. E. D. Hudson, Douglas Bly, and Mr. Selpho, of New York; Mr. Douglas, of Springfield, Mass.; and Mr. Palmer, of Philadelphia. Patients needing artificial limbs can apply to either of these men, and obtain the amount of pecuniary aid mentioned above.

---

Dr. John C. Cheeseman, an eminent surgeon of New York City, died Oct. 11th, 1862, in the 75th year of his age. He was for many years one of the surgeons to the New York Hospital, and a trustee of the College of Physicians and Surgeons.

---

Hospital Inspection.—Drs. Bowditch and Ellis, of Boston, are already engaged in the work of inspection of our army hospitals, under the direction of Sanitary Commission. We think they are faithful and good men for the work.

---

Editor Medical Examiner:—In reply to a scurrilous article in the September Number of the *Medical Journal*, evidently intended for me, I simply state that self-respect forbids me to write another word in this controversy. Leaving the whole matter to the calm judgment of the enlightened members of the profession. I remain, Mr. Editor, very respectfully,

R. ROSKOTEN.

---

Physician's Hand-Book of Practice for 1862: By William Elmer, M.D. New York: W. A. Townsend, Publisher, 39 Walker Street.

The contents of this pocket memorandum and day-book for the physician, are as follows, viz.: Calender for the year 1863; Classification of Diseases; Dr. Marshall Hall's Ready Method; Medicinal Weights and Measures; Abbreviations of Medical Properties and Remedies; List of Remedial Agents; Balnea Medicata; List of Incompatibles; Remarks on the Writing of Prescriptions; Examples of Extemporaneous Prescriptions; Poisons and their Antidotes; Diagnostic Examination of the Urine; The Pulse:—Table of, and other Characters; Explanation of Signs, etc., to be used in the Record; Names and Addresses, Bills, and Accounts; Record of Practice and Treatment; Obstetric Calender; Obstetric Record, Blanks; Wants and General Memoranda; Nurses.

PHYSICIAN'S POCKET MEMORANDUM: For 1863. By C. H. CLEVELAND, M.D. Cincinnati: BRADLEY & WEBB, Printers.

The contents of this work are as follows: Preface; List of Medicines and Classification; Medicines not Classified; Abbreviations used in Prescriptions; Accidents and Emergencies; Poisons and Antidotes; Post Mortems; Preservation and Embalming; Prescription of Medicines; Signs used in Record of Practice; Memoranda of Practice; General Memoranda.

---

EXPERIMENTS ON THE FORMATION OF INFUSORIA IN BOILED SOLUTIONS OF ORGANIC MATTER, ENCLOSED IN HERMETICALLY SEALED VESSELS, AND SUPPLIED WITH PURE AIR.—Dr. Jeffries Wayman relates (*American Journal of Science*, July, 1862,) some very ingenious and interesting experiments to elucidate this subject.

Pasteur, in his admirable researches on fermentation, has brought forward experimental evidence to show that this process depends upon the presence of minute organisms in the fermenting fluid, and that the source of all such organisms is the atmosphere. In support of this opinion he asserts, that when a fluid containing organic matter in solution is put into a flask, and "boiled two or three minutes," and supplied only with air which has been filtered by passing through a tube heated to redness, and the flask is then hermetically sealed, no fermentation takes place, no organisms are formed, and that the contents remain indefinitely without change. But if the same solution is exposed to the air in its ordinary condition, it becomes filled with various living forms. Out of a large number of experiments prepared in the manner above described, he has not known one to give a different result from that mentioned. He further states that if the neck of the flask is drawn out into a very slender curved tube of several inches in length, the contents boiled, and then allowed to cool without the end of the tube being closed, so that the air enters at the ordinary temperature, and has free access to the interior of the flask, even then no fermentation takes place, and no organisms appear. His explanation of this is, that the air which enters first, meets with the hot steam, and the spores or organisms contained in it are killed; while those which enter the tube later move more slowly, and are deposited on the moist walls of it without entering the body of the flask.

The results of Dr. Wyman's experiments have been quite different, and living organisms have made their appearance, in some instances, where even greater precautions were taken than those mentioned by Pasteur. "The boiled solutions of organic matter made use of," by Dr. W., "exposed only to air in hermetically sealed vessels, and exposed to boiling water, became the seat of infusorial life.

These experiments, Dr. W. observes, "throw but little light on the immediate source from which the organisms in question have been derived. Those who reject the doctrine of spontaneous generation in any of the forms in which it has been brought forward, will ascribe them to spores contained either in the air enclosed in the flask, or in the materials of the solution. In support of this view it may be asserted, that it has been proved by the microscopical investigations of Quatrefages, Robin, Pouchet, Pasteur, and others, that the air contains various kinds of organic matter, consisting of minute fragments of dead animals and plants, also the spores of cryptogamous plants, and certain other forms, the appearance of which, as Quatrefages says, suggests that they are eggs. We have made some examinations of our own on this subject, but it would be unnecessary to give the results in detail. We will simply state, that we have carefully examined the dust deposited in attics, also that floating in the air collected on plates of glass covered with glycerine, and have found in such dust, in addition to the debris of animal and vegetable tissues, which last were by far in the greatest abundance, the spores of Cryptogams, some closely resembling those of Confervoid plants, and with them, but much less frequently, what appeared to be the eggs of some of the invertebrate animals, though we were unable to identify them with those of any particular species. We have also found grains of starch in both kinds of dust examined, to the presence of which Pouchet was the first to call attention. When compared with the whole quantity of dust examined, or even with the whole quantity of organic matter, both eggs and spores may be said to be of rare occurrence. We have not in any instance detected dried animalcules which were resuscitated by moisture, and when the dust has been macerated in water, none have appeared until several days afterwards, until after a lapse of time, when they would ordinarily appear in any organic solution.

"Those who advocate the theory of spontaneous generation, on the other hand, will doubtless find, in the experiments here recorded, evidence in support of their views. While they admit that spores and minute eggs are disseminated through the air, they assert that no spores or eggs of any kind have been actually proved by experiment to resist the prolonged action of boiling water. As regards Vibrios, Bacteriums, Spirillums, etc., it has not yet been shown that they have spores; the existence of them is simply inferred from analogy. It is certain that Vibrios are killed by being immersed in water, the temperature of which does not exceed 200° F. We have found all motion, except the Brownian, to cease even at 180° F. We have also proved by several experiments that the spores of common mould are killed, both by being exposed to steam, and by passing through the heated tube used in the experiments described in this article. If, on the one hand, it is urged that all organisms, in so far as the early history of them is known, are derived from ova, and therefore from analogy, we must ascribe a similar origin to these minute beings whose early history we do not know, it may be urged with equal force, on the other hand, that all ova and spores, in so far as we know anything about them, are destroyed by prolonged boiling: therefore, from analogy we are equally bound to infer that Vibrios, Bacteriums, etc., could not have been derived from ova, since these would all have been destroyed by the conditions to which they have been subjected. The argument from analogy is as strong in the one case as in the other.—*Amer. Jour. Med. Sciences.*

---

Inversion of the Uterus occurring Spontaneously Eighty Hours after Delivery.—Some obstetricians believe that inversion of the uterus is always the result of some cause over which the attendant has control, and that in most cases it is caused by undue traction on the cord. Dr. Radford, however, has clearly established that it may occur spontaneously without any fault of the midwife's, and that it may occur not only immediately after delivery, but also after an interval of some days. Mr. Charles Cowan relates (*Edinburgh Medical Journal*, June, 1862,) a case which is conclusive as to this point.

The subject of it was a lady, 40 years of age, always in the enjoyment of good health, who was delivered with the forceps, after a pretty smart labor of 24 hours' duration, of her first child, a strong healthy boy, at 4 A.M., on Thursday, the 15th November, 1861. The placenta was found in the vagina, ten minutes afterwards, and removed. In about half an hour there was slight hemorrhage, which was easily restrained by the application of cold to the pubic region, which caused the uterus to contract firmly: a little brandy was likewise administered, as I attributed the hemorrhage to the weak state of the patient, ocsioned by the protracted labor, and by my efforts to accomplish delivery with the forceps, which occupied me more than half an hour.

I remained with her half an hour longer; and to satisfy myself that all was right I removed the bandage, and, on manipulating the abdomen, I discovered the uterus firmly contracted, about the size of a cricket ball, in its normal situation. The bandage having been replaced I administered a dose of morphia, and took my leave.

For the first three days after her confinement, we had the patient giving the most satisfactory evidence of speedily attaining convalescence. The pulse becomes natural; the appetite returns; the secretions are normal; the breasts are distended with milk, and the mother rejoices in the prospect of being able to nurse her child. Her sister finding her so well on the Saturday afternoon, (two days and a-half after delivery), returns home, and on the Sunday the patient expresses a wish to get up. What could be more gratifying than this? What could more strongly indicate a rapid restoration to health? So far it appears that all is well; but on Sunday at mid-day, about 80 hours after delivery, a change takes place. Eager to test her strength she leaps out of bed, comes to the ground with some degree of violence, staggers to the fireplace, and falls into a chair in a state of syncope.

There had undoubtedly been mischief, and that of no light character, for from this moment a train of symptoms of the most alarming description followed, becoming hourly more and more serious, until the cause was discovered in the invertion of the uterus, and replacement was accomplished. She now again began to show some slight signs of amendment; and we cannot but conclude that the inversion was occasioned by the hurried leap out of bed, especially as the unfavorable symptoms presented themselves then for the first time; and we have further proof of this in the amelioration of these symptoms, commencing almost as soon as the displacement of the uterus was reduced.—*Amer. Jour. Med. Sciences.*

---

Researches on the Influence of Culinary Salt and Coffee on the Metamorphosis of Tissue.—Culinary salt, according to the researches of C. Voit, is a powerful stimulator of the metamorphosis of tissue; it increases, by means of its physical properties, the capillary circulation of fluids in the organism; it increases the oxidation of albumen, and through this the quantity of urea excreted. Culinary salt is also a true diuretic. In order to excrete the salt from the body, water is required; this water passes always through the kidneys, (the only channel for the excretion of culinary salt in the dog,) and is, if the supply of water from without is limited, abstracted from the tissues.

Voit's experiments with *coffee* on a dog led to the inference that coffee does not, as is usually assumed, diminish the metamorphosis of nitrogenous tissue, and the excretion of urea, but, on the contrary, rather increases these processes. On the whole, the dog appeared to be more lively after the use of coffee. The author made also experiments with *coffein* on frogs, and found it to cause, at first, increased irritability of the nervous system, a tendency to reflex-movements and to tetanic convulsions; later, however, phenomena of paralysis. The pupil becomes dilated; the capillary vessels are filled with blood; the heart's contractions are at first increased, later reduced in frequency, they are arrested during the tetanic paroxysms. The author attributes the principal effects of coffee to its action on the nervous system, not to its influence on the tissue-change. The nervous system being rendered more susceptible, the same exciting cause produces a greater effect. Coffee thus refreshes, Voit thinks, the fatigued body, renders the lassitude less perceptible, and in this manner enables us to endure prolonged exertion. The experiments on the influence of *bodily exercise* (treadwheel) on the tissue-change in the well-known dog lead to the unexpected result, that the excretion of urea was not at all, or only very slightly, increased by bodily labor. Voit infers, therefore, that muscular action does not cause increased decomposition of albuminous substances, while it is accompanied with a greater consumption of fat. As the decomposition of albumen is not the source of the production of force, connected with muscular contraction, Voit is inclined to look for it in the development of electricity.—*Brit. and For. Med. and Surg. Journal*, July, 1862.

---

Varicose Ulcers of the Leg.—In a paper read before the Midland Medical Society, J. H. Houghton, surgeon to the Dudley Dispensary, advocates the use of the flannel bandage suggested by Mr. Hunt, in 1857. Cases looked upon as next to incurable, can be managed, with this treatment, without difficulty, and a speedy cure is effected without confinement, or the patient's relinquishing his usual occupation. The wound is strapped with a few strips of soap-plaster, or dressed with some simple dressing, and the bandage applied by first making one turn round the bottom of the leg, then one under the sole of the foot, over the instep, round the back of the foot, again over the instep, till the lower edge of the bandage passes round the foot at the root of the toes, about two turns more round the foot, and then spirally up the leg to the knee. The roller naturally follows this course, and requires no turn till it reaches the calf; if properly applied, it will lie quite even and remain immovable for an indefinite period. In some cases, the patients walked twice a-week nine and twelve miles to have the leg dressed, and followed their usual occupations all the time. Quinine and iron, sarsaparilla and iodide of potassium, etc., are given internally, where indicated. A material called "domette" seems to be preferable to flannel. The roller must be accurately made two and a-half inches wide, and in one piece, eight yards long. The success of the treatment is confirmed by I. K. Spender and H. Crisp, in the *British Medical Journal.* The former recommends, in the place of the soap-plaster, an ointment containing a large quantity of an alkaline earth, as chalk, spread thickly on lint, or the compound lead-plaster of the Pharmacopœia. Mr. Mitchell, of the Lancaster Infirmary, gives in the *Lancet* the following directions for treating old indolent ulcers. First wash the leg well, then fill the ulcer with finely powdered carbonate of iron, and apply a large linen pad, excluding all moisture; then envelop the whole limb in a starched bandage, for about three weeks. The patient may walk a little every day.—*Amer. Med. Times.*

---

The Liver in Cholera Infantum.—Dr. J. Lewis Smith, Curator of the Nursery and Child's Hospital, has published (*American Medical Times*, Sept. 20th, 1862,) some interesting investigations which confirm the prevalent belief that the liver plays an insignificant part in the pathology of the summer complaint of children. "The green stools," he justly remarks, "which have long been referred to the biliary secretion, must be mainly due to causes operating in the intestines, for I have repeatedly noticed that the green color does not appear till we reach the lower part of the jejunum, or upper part of the ileum. Examined under the microscope the green matter is found to be in little fragments or masses, as if produced in the crypts of the intestines."

Dr. S. gives the result of the *post mortem* appearance of the liver in 37 cases. No evidence is afforded by these of any congestion, or torpidity, or hyperactivity, or perverted secretion, or abnormal size of the liver. "In most of the cases the liver was examined microscopically, and the only fact worthy of note observed was the variable amount of fatty matter. Sometimes it was in excess, sometimes in moderate quantity, or rather deficient, and sometimes, apparently, in greater amount in one portion of the organ than in others."

---

MEDICAL COUNCIL OF GREAT BRITAIN.—The doings of this council, from which so much was expected, seems not to have been satisfactory. Its attempts to regulate and improve medical education, and to purge the profession from unworthy members, have been failures; and its new pharmacopœia, which has cost heavily in time and money, is now under protest, in consequence of the council committing the blunder of introducing new weights and measures, which it is contended would create great confusion.

---

### A DOLLAR THAT PAYS WELL.

One of the best seasonable enterprises, now before the public, is that of the Publisher of the *American Agriculturist.* He has secured for his subscribers fine colored editions of the splendid Maps of localities of great interest. One of these covering a space of more than *ten square feet*, shows the entire State of VIRGINIA, so completely that every county, town, city, village, river, brook, mountain, hill, and principal road is readily found. It also embraces the principal parts of Maryland and Pennsylvania. The other Map, covering about fifteen square feet, gives all the Southern or Slave States, including Missouri, Kentucky, Virginia, Maryland, Delaware, and all south of them. Though not so minute as the Map of Virginia, this shows all the counties, principal towns, rivers, etc., of the Southern States. Any person subscribing now for the *Agriculturist*, is presented with a choice of the above two Maps. In addition to this, every new subscriber for 1863, (Vol. 22,) receives the *Agriculturist* for the rest of the year *without charge.* We have long received the *Agriculturist*, and can testify to its real merits. Every number is well illustrated, and contains a very large amount of really useful, practical, reliable information for the Farm, the Garden, and the Household, including a very interesting department for the little ones. No one can fail to get many dollars worth of useful hints from a volume of the *Agriculturist*, while the maps now are so much extra. We have sent for two copies of the paper so as to get both maps.—Send for the paper on our recommendation, or if you prefer, send a dime for a single copy, and examine it for yourself. The address of the Publisher is ORANGE JUDD, 41 Park Row, New York.

